# The glare illusion in individuals with schizophrenia

**DOI:** 10.1016/j.scog.2025.100366

**Published:** 2025-05-19

**Authors:** Hideki Tamura, Aiko Hoshino

**Affiliations:** a Department of Computer Science and Engineering, Toyohashi University of Technology, Japan; b Graduate School of Medicine, Nagoya University, Japan

**Keywords:** Schizophrenia, Visual illusion, The glare illusion, Human psychophysics, Brightness

## Abstract

Individuals with schizophrenia are known to display unique reactions to visual illusions, and prior research has indicated a potential link between their increased susceptibility to geometric illusions and specific symptom profiles. While various illusory experiences have been examined among individuals with schizophrenia, their responses to brightness-related illusions remain poorly understood. In this study, we investigated how individuals with schizophrenia perceive the glare illusion, in which the apparent brightness of the central region is increased. A total of 30 patients with schizophrenia and 34 control participants were recruited. During each trial, a glare or control image (standard stimulus) was presented alongside a control image (comparison stimulus) with one of seven luminance levels. In the glare condition, the standard stimulus was a glare image; in the control condition, two control images were presented, but only the luminance of the comparison stimulus varied. The participants were asked to judge which central region appeared brighter. The results revealed that individuals with schizophrenia exhibited greater susceptibility to the glare illusion than did the control participants. However, no significant associations were found between susceptibility to the glare illusion and scores assessing symptom severity. These findings suggest that differences in visual processing in patients with schizophrenia may increase their susceptibility to brightness illusions, although this phenomenon is independent of symptom characteristics. This information may provide a basis for exploring illusion susceptibility as a potential behavioral index for distinguishing between individuals with schizophrenia and control participants.

## 1 Introduction

Visual illusions serve as valuable tools for understanding perception and cognition, as well as the mechanisms of the visual system. Because the processing of visual illusions involves both bottom-up and top-down mechanisms, these illusions may offer unique opportunities to derive quantifiable behavioral indices that reflect differences in the underlying neural mechanisms. Various visual illusions have been employed to examine differences in visual processing in patients with schizophrenia (Costa et al., 2023b; King et al., 2017; Kogata and Iidaka, 2018; Notredame et al., 2014). Among these, geometric illusions, motion illusions, and depth inversion illusions have been widely studied. Costa et al. proposed classifying visual illusions by the perceptual domain (e.g., geometry, brightness, and motion) and underlying mechanisms (e.g., bottom-up or top-down), clarifying which illusions may best capture altered processing in individuals with schizophrenia (Costa et al., 2023b). In particular, the Müller–Lyer illusion, which distorts the perceived length of lines depending on the orientation of arrow-like fins, and the facial depth inversion illusion, where concave masks are perceived as normal convex faces, have been shown to be processed differently by individuals with schizophrenia and controls, suggesting weakened top-down processing. Susceptibility to the Müller–Lyer illusion has been shown to vary with the stage of schizophrenia across multiple studies (Costa et al., 2023a; Parnas et al., 2001; Rund et al., 1994), further highlighting the growing interest in visual illusions as potential biomarkers for early diagnosis and quantifiable behavioral indices.

Thus, numerous studies have investigated the relationships between schizophrenia and various types of visual illusions, including motion illusions, geometric–optical illusions, illusory contours, and depth inversion illusions, based on the classification approach developed by Costa et al. (2023b). However, brightness illusions remain largely unexplored in schizophrenia research, despite brightness perception being a fundamental aspect of early visual processing. Previous studies have examined simultaneous brightness contrast, in which the perceived brightness of a central region is modulated by the luminance of the surrounding area, in patients with schizophrenia (Grzeczkowski et al., 2018; Kaliuzhna et al., 2019; Yang et al., 2013); however, the findings have been inconsistent. This inconsistency may stem from differences in experimental paradigms or individual variations in contrast sensitivity. Moreover, brightness illusions that rely primarily on local contrast differences and early-stage visual mechanisms may sometimes produce more subtle perceptual effects, which could make the detection of group-level differences more difficult under certain experimental conditions. These findings highlight the need to further investigate brightness illusions that induce stronger perceptual alterations, such as the glare illusion. In contrast to the simultaneous brightness–contrast illusion, the glare illusion increases the perceived brightness beyond what is predicted by local contrast mechanisms, suggesting that additional visual processing mechanisms, possibly involving contextual or top-down modulation, may contribute to this effect. Nevertheless, the perception of brightness illusions in patients with schizophrenia has not yet been thoroughly investigated.

**Fig. 1:**
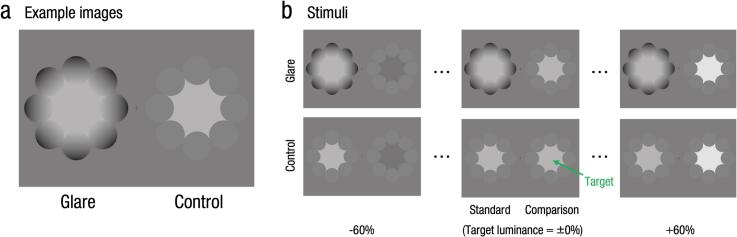
Stimuli. (a) Representative images used in the glare illusion experiments. The left image represents a glare image, whereas the right image represents a control image. The luminance values of the central region in both images are identical. (b) Experimental stimuli. The upper row displays the stimuli used in the glare condition, and the lower row shows the stimuli used in the control condition. Examples of luminance pairs at −60 %, ±0 %, and + 60 % are shown from left to right.

To address this gap, we investigated how individuals with schizophrenia perceive the glare illusion—a phenomenon in which a central region appears brighter due to a surrounding luminance gradient, despite identical physical luminance. As illustrated in Fig. 1a, when a pattern with a luminance gradient in the surrounding region (glare image) is compared to a pattern without a gradient (control image), the central region of the glare image is perceived as being significantly brighter, despite both images having the same physical luminance level (Agostini and Galmonte, 2002; Suzuki et al., 2019b; Suzuki et al., 2019a; Tamura et al., 2016; Zavagno, 1997). Various geometric patterns have been reported to induce the glare illusion, including squares (Agostini and Galmonte, 2002; Zavagno, 1997; Zavagno et al., 2017), circles (Hanada, 2012; Suzuki et al., 2019b; Tamura et al., 2016), patterns composed of multiple circles (Istiqomah et al., 2022; Kinzuka et al., 2021; Suzuki et al., 2019a), and patterns composed of multiple geometric shapes (e.g., Asahi illusion) (Durand et al., 2024; Laeng et al., 2018; Laeng and Endestad, 2012). Despite their differences, these patterns share a common characteristic: the presence of a surrounding luminance gradient that modulates perceived brightness. Moreover, in addition to the increase in the perceived brightness of the central region, the central area has been reported to be perceived as glowing (Tamura et al., 2016) or even dazzling (Hanada, 2012, Hanada, 2023). In addition to subjective evaluations measured through psychophysical experiments, the physiological responses associated with viewing the glare illusion have been investigated. For example, pupil measurements have shown that pupillary constriction occurs more strongly when the glare illusion is observed (Durand et al., 2024; Istiqomah et al., 2022; Laeng et al., 2018; Laeng and Endestad, 2012; Suzuki et al., 2019b; Suzuki et al., 2019a; Zavagno et al., 2017). Furthermore, a study examining the glare illusion in individuals with autism spectrum disorder (ASD) reported that both individuals with ASD and control participants presented similar subjective increases in brightness and pupillary constriction (Laeng et al., 2018). Given that both ASD and schizophrenia have been associated with atypical visual perception, including differences in sensory integration and perceptual organization (Simmons et al., 2009), individuals with schizophrenia may also exhibit altered brightness perception due to differences in visual information processing mechanisms.

Therefore, the aims of this study were to investigate how individuals with schizophrenia perceive the glare illusion and whether their susceptibility to this illusion differs from that of controls. A psychophysical experiment was conducted in which individuals with schizophrenia (SZ) and control participants (CN) were both asked to report which of the two presented images—the glare image or the control image—appeared brighter. The susceptibility to the illusion was then compared between the two groups. Given that individuals with SZ exhibit increased susceptibility to geometric illusions (Costa et al., 2023a), we hypothesized that their altered visual information processing may lead to increased susceptibility to the glare illusion, a type of brightness illusion. Additionally, we examined whether susceptibility to the glare illusion correlates with subjective brightness perception in daily life, which may reflect how everyday visual experiences shape individual differences in illusion perception. Furthermore, we examined whether susceptibility to the glare illusion was related to symptom severity in the SZ group, as previous studies have suggested such links for other illusions. Clarifying this relationship may help determine whether individual differences in illusion perception reflect the clinical characteristics.

## 2 Methods

### 2.1 Participants

A total of 30 participants with schizophrenia (SZ) and 34 control participants (CN) participated in the experiment. The mean age and standard deviation (SD), as well as the sex ratio, for each group were as follows: SZ group, 52.83 ± 8.6 years, with 70 % males; and CN group, 46.38 ± 8.48 years, with 32 % males. The sample size was determined to ensure that at least 30 individuals were included in each group. In prior research on SZ and visual illusions (Costa et al., 2023a), the sample consisted of 14 patients with chronic SZ and 24 controls, indicating that the sample size in this study was reasonable. The SZ group included outpatients with chronic conditions receiving standard treatment, primarily pharmacotherapy, psychotherapy, and rehabilitation. Additionally, the Brief Psychiatric Rating Scale (BPRS) and the Global Assessment of Functioning (GAF) scores of the individuals in the SZ group were measured by the medical staff responsible for each patient. The means and SDs of these scores were 34.77 ± 4.06 and 46.43 ± 12.25, respectively. All procedures were approved by the Research Ethics Committee of the Graduate School of Medicine, Nagoya University, Japan (authorization number: 2023-0406), in 2024, and all ethical standards established in the Declaration of Helsinki were followed. All participants provided informed consent prior to participating in the study.

### 2.2 Apparatus

The experimental stimuli were presented on the display of a Surface Pro 8 (2880 × 1920 resolution, 60 Hz refresh rate). The monitor was calibrated using a spectroradiometer (SR-3AR, TOPCON), ensuring a gamma value of 1. The participants observed the tablet display, which was placed on a table, while seated in a chair. They were instructed to maintain a viewing distance of 50 cm from the display. The experiment was conducted in an indoor environment, with the room lights turned on and sunlight blocked to ensure that no external light directly illuminated the monitor or the participants. The room was one in which participants were accustomed to spending time during daily activities, providing a natural yet controlled environment. The average ambient illuminance measured near the participant at the beginning of the experiment across all participants was 598.94 lx. The experimental script was executed using MATLAB R2024a and Psychtoolbox 3.0 (Brainard, 1997; Kleiner et al., 2007; Pelli, 1997).

### 2.3 Stimuli

Fig. 1 shows the experimental stimuli, namely, glare illusion patterns composed of eight circles (Kinzuka et al., 2021; Suzuki et al., 2019a). Two types of images were prepared: glare images and control images (Fig. 1a). In the glare images, the luminance gradient of the surrounding inducers increased from the outside (darker area, $Y=0.74\;\mathrm{cd}/m^{2}$) to the inside of the image (brighter area, $Y=82.09\;\mathrm{cd}/m^{2}$, defined as the reference luminance). In the control images, the luminance gradient of the inducers was removed and replaced with a uniform luminance equal to the average luminance of the inducer. Thus, the average luminance of the inducers in the glare and control images was identical. The luminance of the central region in the control images was set to seven levels relative to the target luminance of $Y=82.09\;\mathrm{cd}/m^{2}$, corresponding to −60 %, −40 %, −20 %, ±0 %, +20 %, +40 %, and + 60 %, forming the luminance factor (the −60 %, ±0 %, +60 % examples are shown in Fig. 1b). Each image was presented with an inscribed circle in the central region, subtending the visual angle by 5 degrees.

### 2.4 Procedure

Each participant was instructed to sit at a distance of 50 cm from the tablet screen at the start of the experiment. This viewing distance was applied consistently across all participants to ensure comparable visual conditions. No physical restraints, such as a chin rest, were used to reduce the participants' burden. For participants in the schizophrenia group, an occupational therapist assisted with the procedure to ensure their comfort and understanding. Although minor variation in the viewing distance may have occurred due to differences in posture or body height, we considered such differences to be minimal and unlikely to have systematically affected the results.

After a fixation point was presented at the center of the screen for 1 s, the stimuli were simultaneously displayed on both sides of the fixation point. In the glare condition, the pair consisted of a glare image (standard stimulus) and a control image (comparison stimulus). In the control condition, the pair consisted of a control image (standard stimulus) and another control image (comparison stimulus). For the standard stimulus, the luminance of the central region was fixed at $Y=82.09\;\mathrm{cd}/m^{2}$; this stimulus was a glare image in the glare condition and a control image in the control condition. The comparison stimulus was always a control image, and the luminance of the central region of this image was set to one of the seven levels defined by the luminance factor. The control condition was included to estimate the baseline performance in brightness perception using nonillusory stimuli and to control for individual differences in brightness discrimination ability. This approach allowed for a more accurate quantification of the illusion-induced perceptual bias.

While fixating on the fixation point, the participants compared the central regions of the two images presented and responded by touching the image they perceived as brighter. After the participant provided a response, the next trial began. The stimuli were displayed until a response was provided. The left and right positions of the standard and comparison stimuli were randomized. Each participant completed 112 trials (2 conditions × 7 luminance levels × 2 left–right positions × 4 repetitions), and the trial order was randomized. A break was provided every 28 trials, and the participants were allowed to rest for as long as they needed.

Afterward, the participants completed a questionnaire, which was provided to assess their subjective evaluations of daily life. Among the questions, three items specifically addressed brightness perception, and participants were asked to respond using a 5-point Likert scale: Q1—I find car headlights to be excessively bright; Q2—I find sunlight to be excessively bright; and Q3—My eyes tend to feel fatigued during dim light conditions in the evening. The responses were rated as follows: 5 = strongly agree, 4 = agree, 3 = neutral, 2 = disagree, and 1 = strongly disagree. The purpose of including these brightness-related questions was to explore whether general sensitivity to brightness in everyday contexts, such as visual fatigue or discomfort, might be related to susceptibility to the glare illusion. Although the illusion depends on the spatial configuration, we hypothesized that individuals who are more sensitive to brightness in general may also perceive the glare illusion more strongly.

### 2.5 Data analysis

The data were analyzed using R (version 4.3.3). For each participant, the probability of responding that the comparison stimulus appeared brighter was calculated at each luminance level. The response probabilities across the seven luminance levels for each pattern condition were fitted with a probit function to derive the psychometric functions for each condition. Additionally, the point of subjective equality (PSE) was calculated from these functions.

Data were excluded if the participant's responses could not be adequately fitted with a probit function, which we interpreted as indicative of a failure to understand the task or respond consistently. Specifically, data were removed if either the glare or control condition met one or more of the following criteria: (1) the participant never responded that the comparison stimulus appeared brighter; (2) the estimated PSE was extremely negative and outside the stimulus range; or (3) the JND could not be computed because some of the parameters required for its calculation were estimated as NaN. As a result, 6 participants from the SZ group and 3 participants from the CN group were excluded. The remaining data from the 24 individuals in the SZ group and 31 participants in the CN group were used for subsequent analyses.

The PSE values were predicted using a linear mixed-effects model to examine the presence of brightness enhancement effects induced by the glare illusion. The model included the fixed effects of the group (G: SZ vs. CN) and condition (C: glare vs. control), the interaction between the group and condition with an intercept, and a random effect of the participants. Thus, the model was specified as follows: $\mathrm{PSE}=G+C+G:C+1+\left(1|\mathrm{ParticipantID}\right)$. Post hoc pairwise comparisons were conducted with the Bonferroni adjustment for *p* values.

Following the approach of Costa et al. (2023a), susceptibility to the illusion was defined as the PSE difference, which was calculated as the difference between the PSE values in the glare and control conditions. This value was computed for all participants, and differences between the SZ and CN groups were tested using Welch's two-sample *t-*test.

In addition, pairwise correlations among the subjective brightness responses (Q1, Q2, and Q3), participants' ages, BPRS scores, and GAF scores were analyzed.

## 3 Results

**Fig. 2:**
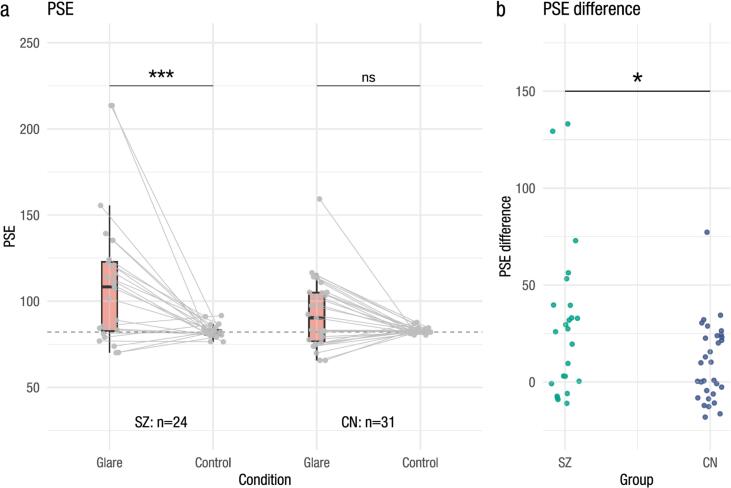
Experimental results. (a) PSE values for each condition. The boxplot displays the distribution, with the data points for individual participants overlaid. The horizontal dashed line represents the target luminance (Y = 82.09 cd/m^2^). (b) Differences in the PSE values among the groups. Each dot represents the PSE value for an individual participant.

Fig. 2 shows the experimental results (see Fig. S1 for the psychometric functions of individual participants and condition-wise averages for each group). Fig. 2a shows the PSE values, and the mean PSE values and SDs for each group and condition pair were 111.61 ± 39.18 and 82.52 ± 3.68 (glare and control conditions in the SZ group) and 92.87 ± 20.07 and 82.73 ± 1.60 (glare and control conditions in the CN group), respectively. The results of the linear mixed-effects models (LMMs; see Table 1) revealed a significant main effect of the stimulus condition, indicating that the glare stimulus was perceived as brighter than the control stimulus ($\hat{\beta }=-29.08$, 95 % CI $\left[-41.04,-17.13\right]$, $t\left(53.00\right)=-4.77$, p<.001, Cohen's d=−1.38). Additionally, a significant main effect of the group was observed, with the individuals in the SZ group perceiving the stimuli as brighter than the individuals in the CN group ($\hat{\beta }=-18.74$, 95 % CI $\left[-30.05,-7.43\right]$, $t\left(105.99\right)=-3.25$, p=.002, Cohen's d=−0.89). Furthermore, a significant interaction effect was observed ($\hat{\beta }=18.94$, 95 % CI $\left[3.0234.87\right]$, $t\left(53.00\right)=2.33$, p=.024, Cohen's d=0.90).

Post hoc tests revealed that the individuals in the SZ group perceived the glare stimuli as brighter than the control stimuli (ΔM=29.08, 95 % CI_Bonferroni(6)_ $\left[12.3645.80\right]$, $t\left(53\right)=4.77$, *p*_Bonferroni(6)_ < 0.001). In contrast, no significant difference between the perception of the glare and control stimuli was observed among the individuals in the CN group (ΔM=10.14, 95 % CI _Bonferroni(6)_ $\left[-4.57,24.85\right]$, $t\left(53\right)=1.89$, *p*_Bonferroni(6)_ = 0.386). The complete comparison results are presented in Table 2.

**Table 1:** Summary of the parameters of the LMMs based on the PSE values.

Term	$\hat{\beta }$	95 % CI	*t*	*df*	*p*	Cohen's *d*
Intercept	111.61	[103.11, 120.10]	25.75	105.99	< 0.001	5.28
Stimulus	−29.08	[−41.04, −17.13]	−4.77	53.00	< 0.001	−1.38
Group	−18.74	[−30.05, −7.43]	−3.25	105.99	0.002	−0.89
Stimulus × Group	18.94	[3.02, 34.87]	2.33	53.00	0.024	0.90

**Table 2:** Post hoc comparisons with *t-*tests and Cohen's d values for each pair.

Contrast	Δ*M*	95 % CI_Bonferroni(6)_	*t*	*df*	*p*_Bonferroni(6)_	Cohen's *d*
Glare SZ – Control SZ	29.08	[12.36, 45.80]	4.77	53	< 0.001	0.65
Glare SZ – Glare CN	18.74	[3.22, 34.26]	3.25	105.99	0.009	0.31
Glare SZ – Control CN	28.88	[13.36, 44.40]	5.00	105.99	< 0.001	0.48
Control SZ – Glare CN	−10.34	[−25.86, 5.18]	−1.79	105.99	0.456	−0.17
Control SZ – Control CN	−0.20	[−15.73, 15.32]	−0.04	105.99	> 0.999	0.00
Glare CN – Control CN	10.14	[−4.57, 24.85]	1.89	53	0.386	0.26

Fig. 2b shows the susceptibility of individuals to the glare illusion (PSE difference), which was significantly greater among the individuals with SZ than among the individuals in the CN group (ΔM=18.94, 95 % CI $\left[1.1236.76\right]$, $t\left(32.58\right)=2.16$, p=.038, Cohen's d=0.61). This finding indicates that individuals with SZ were more likely to perceive the glare illusion as brighter than the participants in the CN group.

Notably, no significant gender differences were observed in the PSE difference across all participants (ΔM=−15.00, 95 % CI $\left[-31.67,1.67\right]$, $t\left(44.41\right)=-1.81$, p=.077, Cohen's d=−0.49), within the schizophrenia group (ΔM=−28.26, 95 % CI $\left[-59.14,2.63\right]$, $t\left(15.94\right)=-1.94$, p=.070, Cohen's d=−0.75), or within the control group (ΔM=4.84, 95 % CI $\left[-9.42,19.10\right]$, $t\left(24.83\right)=0.70$, p=.491, Cohen's d=0.24).

**Fig. 3:**
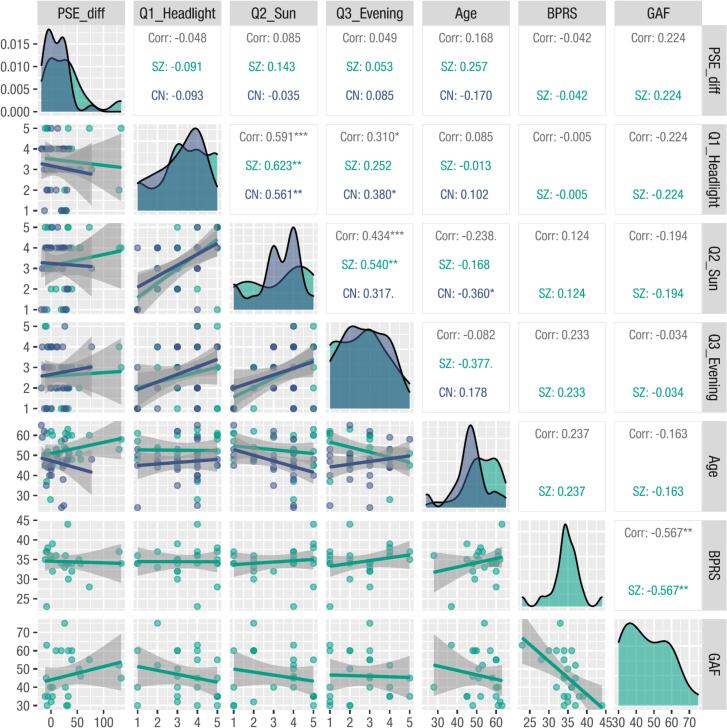
Plots showing the distributions and correlations among pairs of variables. The diagonal matrix displays the distributions of the PSE difference, Q1 response, Q2 response, Q3 response, age, BPRS score, and GAF score for each group. The lower triangular matrix presents scatter plots of variable pairs, including regression lines and their confidence intervals. The upper triangular matrix shows the correlation coefficients between variable pairs (from top to bottom: overall, SZ group only, and CN group only). The asterisks and dots indicate significant differences: . < 0.1, * < 0.05, ** < 0.01, and *** < 0.001.

As shown in Fig. 3, no significant correlations were observed between the PSE difference and the results of the subjective questionnaire for all participants (PSE difference and Q1: $r\left(53\right)=-.048$, 95 % CI $\left[-.309,.220\right]$, p=.727; PSE difference and Q2: $r\left(53\right)=.085$, 95 % CI $\left[-.184,.343\right]$, p=.536; PSE difference and Q3: $r\left(53\right)=.049$, 95 % CI $\left[-.219,.310\right]$, p=.723). Similarly, no significant correlation was found between the PSE difference and the age of the participants ($r\left(53\right)=.168$, 95 % CI $\left[-.102,.415\right]$, p=.219). Additionally, among the individuals with SZ, no significant correlations between the PSE difference and the BPRS or GAF scores were observed (PSE difference and BPRS: $r\left(22\right)=-.042$, 95 % CI $\left[-.438,.368\right]$, p=.847; PSE difference and GAF: $r\left(22\right)=.224$, 95 % CI $\left[-.197,.575\right]$, p=.293). Note that these null findings should be interpreted with caution, as the statistical power to detect small effects was limited (estimated power < 0.3 for the correlations examined above). On the other hand, significant correlations were observed among the responses on the three subjective questionnaire items among all participants (Q1 and Q2: $r\left(53\right)=.591$, 95 % CI $\left[.386.740\right]$, p<.001; Q2 and Q3: $r\left(53\right)=.434$, 95 % CI $\left[.190.627\right]$, p<.001; and Q1 and Q3: $r\left(53\right)=.310$, 95 % CI $\left[.049.532\right]$, p<.05).

## 4 Discussion

In this study, we investigated the perception of the glare illusion in individuals with schizophrenia (SZ) using a psychophysical experiment. A comparison of the PSE values confirmed the increase in the brightness effect consistently documented in previous glare illusion studies, with glare images being perceived as brighter than control images. However, this effect was observed only among the individuals with SZ, whereas no significant difference between the perceived brightness of the glare and control images was observed in the control (CN) group. A primary contribution of this study is the finding that susceptibility to the glare illusion (PSE difference) was significantly greater among individuals with SZ than among participants in the CN group. This finding suggests that altered visual information processing in individuals with SZ may influence brightness perception. However, susceptibility to the glare illusion was not found to be correlated with subjective brightness perception in daily life, age, or disease severity. These results indicate that while individuals with SZ were more likely to perceive the glare image as brighter than the control image, this increased susceptibility was not associated with the severity of their symptoms. These findings suggest that SZ may affect geometric and brightness illusions through distinct mechanisms.

One possible explanation for the increased brightness perception among individuals with SZ is that cumulative alterations in visual processing, such as differences in contrast sensitivity and sensory gating, may lead to increased susceptibility to the glare illusion. Although no significant correlations were found between illusion susceptibility and symptom scores within the schizophrenia group, the observed group-level differences suggest that altered visual processing may occur independently of specific symptom characteristics. This result implies that perceptual differences can emerge at the group level even in the absence of strong individual-level associations.

Considering previous theories on the mechanisms underlying the glare illusion, differences in either bottom-up or top-down processing may have led to a compensatory shift in the reliance on the other mechanism, making individuals with SZ more prone to perceiving the illusion as brighter. First, a reduction in bottom-up processing efficiency (Luo et al., 2021) could increase the reliance on top-down processing, leading to greater susceptibility to the illusion. Specifically, individuals with SZ exhibit differences in the early stages of visual processing, such as reduced contrast sensitivity (Kantrowitz et al., 2009) and altered sensory gating responses (Ludewig et al., 2003; Preuss et al., 2011). These deficits may increase the reliance on top-down interpretations of light diffusion based on prior experience, ultimately leading to increased susceptibility to brightness illusions.

SZ is also associated with differences in top-down processing (Berkovitch et al., 2018; Silverstein et al., 2006), which may increase the reliance on bottom-up processing when the glare illusion is observed. In this case, the luminance gradient of the inducing stimulus, a key element of the illusion (Hanada, 2012, Hanada, 2023), might have strongly influenced low-level visual processing, further increasing the effects associated with the illusion. This finding may reflect broader differences in contextual modulation in patients with SZ. For example, Tadin et al. reported weakened center–surround suppression in motion perception (Tadin et al., 2006), suggesting that atypical contextual integration may also underlie increased susceptibility to the glare illusion. Additionally, studies on brightness induction have yielded conflicting findings regarding the visual mechanisms involved in brightness perception (retina, lateral geniculate nucleus, and primary visual cortex) in patients with SZ. While some studies have reported relatively typical processing (Yang et al., 2013), other studies have suggested variations in these mechanisms (Delord et al., 2006). Given these inconsistencies, further research is needed to determine how alterations in bottom-up and top-down processing contribute to brightness perception in patients with SZ. Understanding these mechanisms may provide insights into the broader differences in visual perception associated with this disorder.

Although a main effect of the pattern type (glare vs. control) was observed across all participants, an analysis within the CN group did not reveal a significant difference between the perceptions of the glare and control images (Fig. 2a, right panel). This finding differs from those of previous studies on subjective brightness perception in the glare illusion, which consistently reported that glare images are perceived as brighter than control images are (e.g., Tamura et al., 2016). One possible explanation for this discrepancy is the age difference between the control group in this study and the participants in previous studies. Specifically, age-related factors may have influenced the perception of the glare illusion. Most previous studies on the perception of the glare illusion have focused primarily on participants in their twenties, whereas the average age of the individuals in the control group was greater in the present study. These findings suggest that aging may modulate or diminish the brightness enhancement effect associated with the glare illusion. However, the post hoc power analysis indicated that the statistical power to detect this difference within the control group was limited (power = 0.283); therefore, this null result should be interpreted with caution.

This study has several limitations. First, although we focused on behavioral responses, pupillary data may also play a role in the perception of such illusions, as suggested by previous studies (e.g., Kinzuka et al., 2021; Suzuki et al., 2019a). Laeng et al. reported that pupillary constriction occurred in individuals with ASD while observing the glare illusion (Laeng et al., 2018). Thus, individuals with SZ may exhibit similar pupillary responses to the glare illusion, which should be further explored. Second, while brightness perception was increased among individuals with SZ, whether previously reported glow and dazzling perceptions are also affected by SZ symptoms remains unclear (Tamura et al., 2016). Previous research has examined not only which stimulus appears brighter but also which stimulus appears more dazzling in the glare illusion (Hanada, 2012, Hanada, 2023). In future studies, new insights could be provided by directly assessing how individuals with SZ perceive glare. Third, given the high heterogeneity of SZ symptoms among patients (Tandon et al., 2013), further studies are needed to explore the relationship between the susceptibility to brightness illusions and other psychiatric conditions.

## 5 Conclusion

In this study, we investigated how individuals with SZ perceive the glare illusion. The results showed that they were more susceptible to the illusion than were the control participants. However, this increased sensitivity was not associated with symptom severity or subjective brightness perception in daily life. These findings suggest that altered visual processing may underlie this susceptibility, independent of clinical symptoms or everyday experiences. This enhanced illusion susceptibility may also serve as a potential behavioral index for distinguishing individuals with schizophrenia from control participants. Further research is needed to clarify the underlying mechanisms and the role of contextual factors in brightness perception.

The following is the supplementary data related to this article.

Figure S1 Psychometric functions of individual participants and condition-wise averages for each group. The xaxis represents the luminance of the central region of the comparison stimulus, and the y-axis indicates the probability of judging the comparison stimulus as brighter. The colors denote the stimulus conditions; the thick lines represent the group-averaged psychometric functions, and the thin lines correspond to individual participants. The left panel shows data from the schizophrenia (SZ) group, and the right panel shows data from the control (CN) group. Note that the group-averaged psychometric curves in this figure were fitted to the mean response probabilities at each luminance level. In contrast, the PSEs reported in the Results and Fig. 2 were calculated by averaging the individually estimated PSEs. Therefore, the curve positions may differ from the average PSEs shown elsewhere. Figure S1

## CRediT authorship contribution statement

**Hideki Tamura:** Writing – review & editing, Writing – original draft, Visualization, Validation, Software, Resources, Project administration, Methodology, Investigation, Funding acquisition, Formal analysis, Data curation, Conceptualization. **Aiko Hoshino:** Writing – review & editing, Writing – original draft, Validation, Resources, Project administration, Methodology, Investigation, Funding acquisition, Data curation, Conceptualization.

## Declaration of Generative AI and AI-assisted technologies in the writing process

During the preparation of this work, the authors used ChatGPT 4o to improve the language, and the manuscript has been proofread by native English speakers through an English editing service. After using the tool and service, the authors reviewed and edited the content as needed and take full responsibility for the content of the publication.

## Declaration of competing interest

The authors declare that they have no competing interests.

## Data Availability

The data that support the findings of this study are available from the corresponding author upon reasonable request. However, data sharing is subject to restrictions according to the ethical approval obtained for this study, and only deidentified or limited datasets may be shared to ensure participant confidentiality.
