# Supplementary material for: The glare illusion in individuals with schizophrenia
Source: Schizophr Res Cogn. 2025 May 19;41:100366. doi: 10.1016/j.scog.2025.100366 (PMC12148450; doi:10.1016/j.scog.2025.100366)
Supplement: Figure S1 — Psychometric functions of individual participants and condition-wise averages for each group. The xaxis represents the luminance of the central region of the comparison stimulus, and the y-axis indicates the probability of judging the comparison stimulus as brighter. The colors denote the stimulus conditions; the thick lines represent the group-averaged psychometric functions, and the thin lines correspond to individual participants. The left panel shows data from the schizophrenia (SZ) group, and the right panel shows data from the control (CN) group. Note that the group-averaged psychometric curves in this figure were fitted to the mean response probabilities at each luminance level. In contrast, the PSEs reported in the Results and Fig. 2 were calculated by averaging the individually estimated PSEs. Therefore, the curve positions may differ from the average PSEs shown elsewhere. [file mmc1.pdf]

Supplement Information

The glare illusion in individuals with schizophrenia

Hideki Tamura[1] & Aiko Hoshino[2]

[1] Department of Computer Science and Engineering, Toyohashi University of Technology

[2] Graduate School of Medicine, Nagoya University

Correspondence concerning this article should be addressed to Hideki Tamura, 1-1 Hibarigaoka, Tempaku-cho, Toyohashi, Aichi, JAPAN, 441-8580. E-mail: tamura@cs.tut.ac.jp

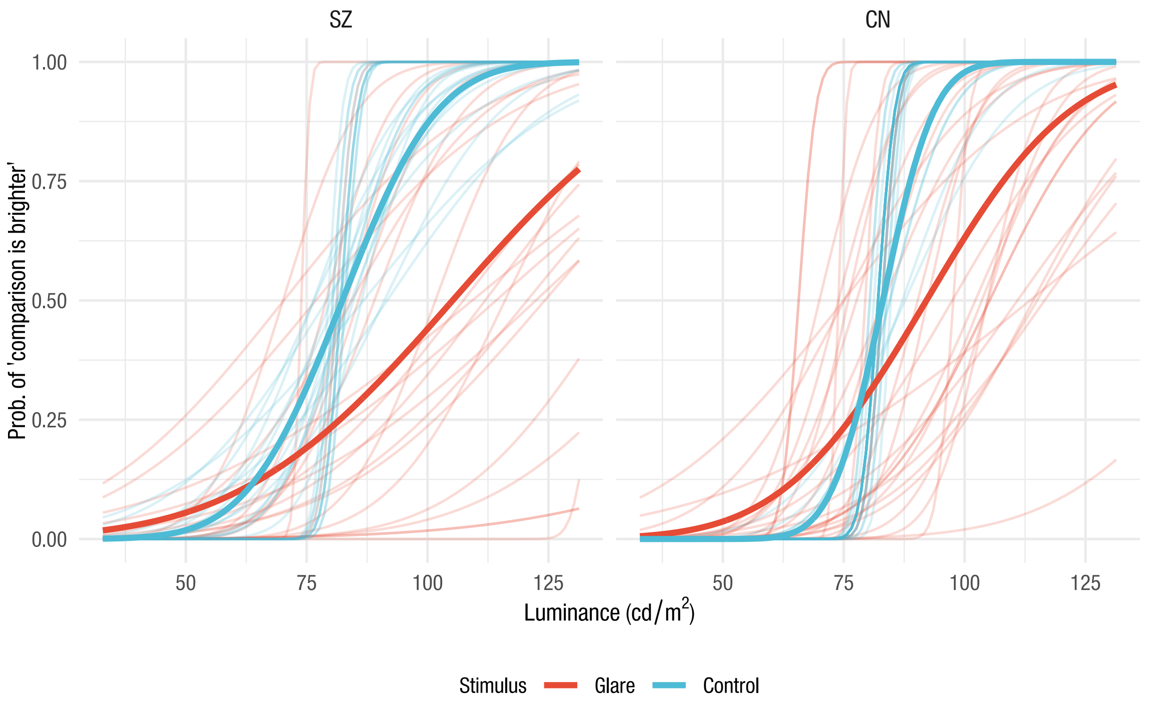

Figure S1

Psychometric functions of individual participants and condition-wise averages for each group. The x-axis represents the luminance of the central region of the comparison stimulus, and the y-axis indicates the probability of judging the comparison stimulus as brighter. The colors denote the stimulus conditions; the thick lines represent the group-averaged psychometric functions, and the thin lines correspond to individual participants. The left panel shows data from the schizophrenia (SZ) group, and the right panel shows data from the control (CN) group. Note that the group-averaged psychometric curves in this figure were fitted to the mean response probabilities at each luminance level. In contrast, the PSEs reported in the Results and Figure 2 were calculated by averaging the individually estimated PSEs. Therefore, the curve positions may differ from the average PSEs shown elsewhere.
